# Changing pattern in the basal ganglia: motor switching under reduced dopaminergic drive

**DOI:** 10.1038/srep23327

**Published:** 2016-03-23

**Authors:** Vincenzo G. Fiore, Francesco Rigoli, Max-Philipp Stenner, Tino Zaehle, Frank Hirth, Hans-Jochen Heinze, Raymond J. Dolan

**Affiliations:** 1 Wellcome Trust Centre for Neuroimaging, University College London, 12 Queen Square, London WC1N 3BG, United Kingdom; 2 Department of Neurology, Otto-von-Guericke-University Magdeburg, Leipziger Str. 44, 39120 Magdeburg, Germany; 3 King’s College London, Institute of Psychiatry, Psychology & Neuroscience, Department of Basic & Clinical Neuroscience, London, UK; 4 Department of Behavioral Neurology, Leibniz Institute for Neurobiology, Brenneckestr. 6, 39118 Magdeburg, Germany; 5 Max Planck UCL Centre for Computational Psychiatry and Ageing Research, 10-12 Russell Square, London, WC1B 5EH, United Kingdom; 6 Centre for BrainHealth, Univesity of Texas at Dallas, 2200 West Mockingbird Lane, Dallas, TX 75235, USA

## Abstract

Action selection in the basal ganglia is often described within the framework of a standard model, associating low dopaminergic drive with motor suppression. Whilst powerful, this model does not explain several clinical and experimental data, including varying therapeutic efficacy across movement disorders. We tested the predictions of this model in patients with Parkinson’s disease, on and off subthalamic deep brain stimulation (DBS), focussing on adaptive sensory-motor responses to a changing environment and maintenance of an action until it is no longer suitable. Surprisingly, we observed prolonged perseverance under on-stimulation, and high inter-individual variability in terms of the motor selections performed when comparing the two conditions. To account for these data, we revised the standard model exploring its space of parameters and associated motor functions and found that, depending on effective connectivity between external and internal parts of the globus pallidus and saliency of the sensory input, a low dopaminergic drive can result in increased, dysfunctional, motor switching, besides motor suppression. This new framework provides insight into the biophysical mechanisms underlying DBS, allowing a description in terms of alteration of the signal-to-baseline ratio in the indirect pathway, which better account of known electrophysiological data in comparison with the standard model.

The basal ganglia (BG) are evolutionarily conserved interconnected nuclei which regulate action selection, initiation and maintenance^1^. Focal lesions and dysfunction of the BG are associated with motoric dysfunctions as seen in Parkinsonism, Huntington’s disease and dystonia, as well as a range of neuropsychiatric disorders^2^. The functions of the BG, as well as disease-related dysfunction, are often explained within an influential, standard model, developed in the 1980’s^3^,^4^,^5^,^6^,^7^. In essence this model describes the basic anatomical and neurochemical characteristics of the BG nuclei and relates movement disorders to imbalances between BG circuitries.

Subsequent elaborations^8^,^9^,^10^,^11^ highlight three competing pathways (Fig. 1), two of which originate from distinct populations of striatal GABAergic medium spiny neurons, each characterised by distinct dopamine (DA) receptor subtypes, whereas the third originate in the glutamatergic neurons of the subthalamic nucleus (STN). This framework assumes the direct pathway, enriched in D1 (excitatory) receptors, facilitates movement and adaptability, whereas the indirect pathway, enriched in D2 (inhibitory) receptors, interacts with the STN (hyperdirct pathway) to control or impede movement.

The attractiveness of the standard model resides in it providing testable predictions and explanations for putative circuit dysfunctions underlying movement disorders, such as Parkinson’s disease. Motor manifestations of Parkinson’s disease typically manifest when DA release in the dorsal striatum decreases 60–70% below baseline^12^. Consistent with reduced DA release, the standard model explains disease-related movement impairments as a decreased influence of the direct pathway and an increased influence of the indirect and hyperdirect pathways, resulting in motor suppression. The standard model predicts that a normal balance between competing BG pathways can be restored by decreasing indirect and hyperdirect pathway activity, for example via lesions of the globus pallidus pars externa (GPe) or the STN.

When Deep Brain Stimulation (DBS) was developed, its effect on behaviour was found very similar to those of a local lesion, so that it was initially assumed the local effect in the stimulated area was that of a reversible suppression of the neural activity^13^,^14^,^15^. For instance, in Parkinson’s disease, DBS of the STN reduces tremor, rigidity, and bradykinesia^16^. However, the mechanism of action of subthalamic DBS is still debated^14^,^17^. Although behavioural improvements are akin to those caused by lesions to the same locus, paradoxically DBS acts by increasing, rather than decreasing (as in lesions), activity of downstream nuclei^18^,^19^. Orthodromic and antidromic pulses generated by DBS increase extracellular levels of both Glu and GABA in BG output nuclei during stimulation of STN, implying increased activity of both STN and GPe^20^,^21^,^22^,^23^. It has also been hypothesised that DBS cancels pathological low frequency activity signature (beta oscillations in the range of 15–25 Hz) associated with BG dysfunctions by imposing a more stable activity pattern^24^,^25^. Both GPe and STN are implicated in generating these oscillations in field potentials under a condition of reduced striatal DA outflow^26^. Together, this evidence suggests that the role of the nuclei of the indirect pathway in mediating effects of striatal DA release on motor output is more complex than predicted in the standard model. A limited understanding of this complexity might impede advances in use of DBS therapies beyond trial and error approaches, whereas a better model of indirect pathway function might explain and, ultimately, help avoid common side effects associated with DBS therapy^27^,^28^,^29^.

Here we assay BG functions in an experiment involving a cohort of twenty-one Parkinson’s disease patients treated with DBS of the STN. Comparing behavioural responses of patients on- and off-stimulation, we show that a classical view of competition between pathways provides an incomplete description of BG function, and fails to predict the entirety of the observed data. To address this problem, we explored the space of parameters offered by a version of the standard neural model grounded on channelled (i.e. representation specific) action selection^9^,^10^,^30^,^31^,^32^,^33^,^34^. Our simulations show that effective connectivity^35^ characterising GPe efferents enables two different functions. On the one hand GPe interacts with the hyperdirect pathway and STN (via long indirect pathway, Fig. 1), facilitating a diffuse inhibition of all actions as postulated in the standard model. On the other hand, GPe regulates oscillatory selections via direct inhibitions towards the output system of the BG (short indirect pathway, Fig. 1), which in the case of the sensorimotor loop is represented by the internal part of the globus pallidus (GPi). We suggest that a balance between these two functions can account for observed behavioural effects defined along two dimensions, namely the time required to adaptively disengage from a selection (perseverance time) and the disposition to change an ongoing selection in favour of initiating a new one, despite lack of changes in the sensory input (frequency of switching). We test model predictions in a second experiment involving healthy volunteers under different pharmacological conditions while they performed a task designed to overactivate the indirect pathway. We expected decrease in disengaging time and increase in the number of unnecessary switching. The behaviour recorded shows trends in keeping with the predictions of our revised model, which we suggest provides an improved theoretical framework for understanding functions and dysfunctions of the BG, casting light on likely mechanisms underlying subthalamic DBS.

## Results

### Experiment 1: action selection on and off subthalamic stimulation

**Figure 2:**
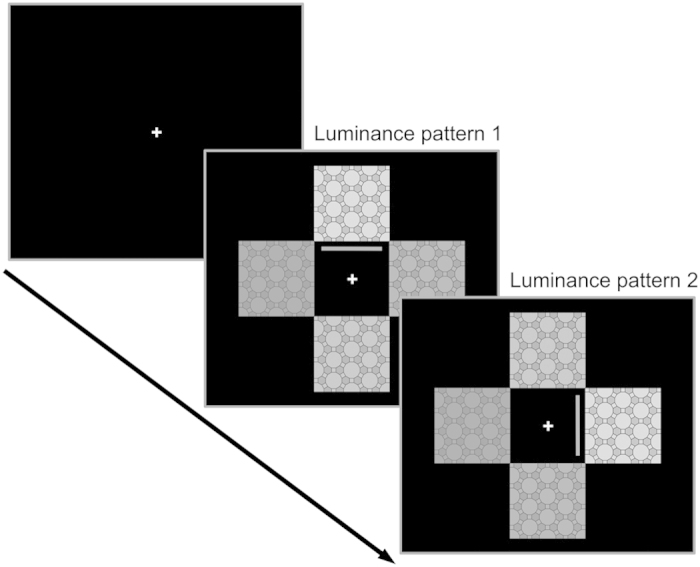
Visual Discrimination Task. Task used for the two behavioural studies. Four black and white images in a cross shape form a series of static luminance patterns that change with a variable interval. The participants are tasked to use the keyboard to choose the image they consider the brightest and then to keep pressing the chosen key until a new luminance pattern is presented and a new selection is necessary. The grey line represents the feedback provided to the participants and, in this illustration of an ideal behaviour, always represents the selection of the correct cue.

We implemented a computer-based task that required simple and quick sensory-motor responses that were either maintained or switched in order to adapt to a changing environment. In brief, patients were required to select the brightest image among four squared tessellation figures in grey scale and, importantly, to maintain this selection by keeping a key pressed until another image became the brightest (Fig. 2). The images were positioned in a cross shape (top, bottom, left and right): we refer to any configuration of the four figures in the given positions as “luminance pattern” throughout this manuscript.

**Figure 3:**
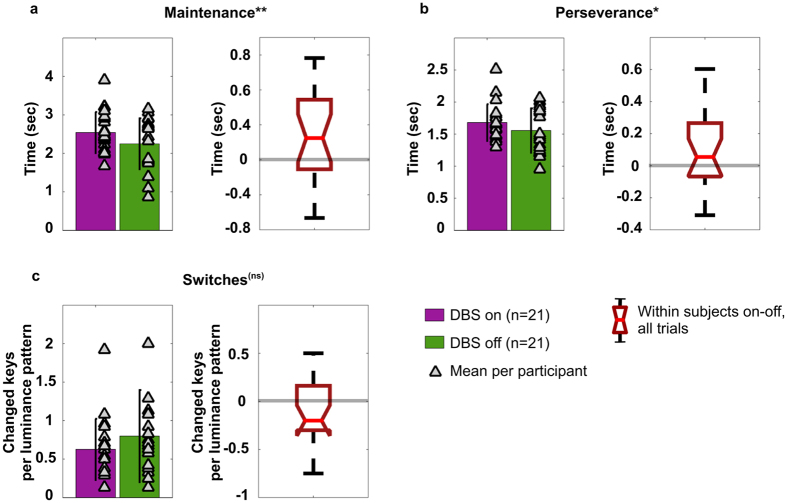
Behavioural indices recorded in Experiment 1. (**a**) maintenance: time spent keeping a key pressed, which shows the treatment significantly increases the ability of the patient to maintain a selection (t(20) = 2.62, p = 0.016); (**b**) perseverance: time required to disengage from a selection after each change of luminance pattern on the screen, which shows the treatment significantly increases the chances the patient will persevere in any selection (t(20) = 2.14, p = 0.045); (**c**) number of changes of selection or switches, per luminance pattern, which does not show a significant effect of treatment (t(20) = −1.4648, p = 0.16). Histograms represent mean measures and standard deviation, per trial, under on- and off- subthalamic stimulation. Triangular markers represent mean values per participant, under each condition. The distribution in quartiles of within subject differences, per measure, is represented in the boxplots. Statistical significance (T test comparisons) expressed as follows: **for p ≤ 0.01; *for 0.05 > p ≥ 0.01; ^(ns)^ for not significant.

The ability to sustain any key selection, and keep any key pressed, was significantly affected by DBS. Patients in the on-stimulation condition maintained their selections for a longer time (on-stimulation: 2542 ms vs off-stimulation: 2249 ms, t(20) = 2.62, p = 0.016, Fig. 3a) than was the case in the off condition. Surprisingly, our perseverance index showed patients are quicker to disengage from a prior selection after a change in luminance pattern when off-stimulation compared to when on-stimulation (on-stimulation: 1680 ms vs off-stimulation: 1556 ms, t(20) = 2.14, p = 0.045, Fig. 3b). The two indices are positively correlated (*r* = 0.63, p = 0.002, sup. Fig. 1a), therefore patients on-stimulation sustain selections as required by the task, but also persevere more. By contrast, patients off-stimulation disengage more easily from their selections after a change in luminance pattern, but display a difficulty maintaining a selection for the requisite time. We have tested whether a measure of perseverance or maintenance could have been biased by a general reduced activity under the off stimulation condition. We found that both the number of times a change in luminance pattern caused no response and the time duration when no key was pressed did not differ as a function of treatment level (p = 0.37, p = 0.45, respectively). In the framework of a standard model, this off-stimulation related deficit in maintenance is explained as a result of greater activity within the indirect pathway (due to low DA release), thereby impairing action. Under this framework, the measure of perseverance is not as easily explained. An efficient direct pathway (as assumed under on-stimulation) is expected to cause quicker action selections and increased motor flexibility in response to changing stimuli.

To measure the effect of DBS on motor flexibility, we analysed the number of “switches” (i.e. changed selections) per trial, considering the task requires one switch per each change of luminance pattern. The competing pathways hypothesis necessarily implies that off-stimulation the overall number of switches produced should decrease. Interestingly, this key prediction of the standard model is not met. Indeed, the difference in the number of switches between on- and off-stimulation fails to reach statistical significance (t(20) = −1.48, p = 0.16, Fig. 3c). Differences in disease severity, measured via the unified Parkinson’s disease rating scale (UPDRS, Table 1: scores of 16 patients are available off stimulation), are not correlated with the high variability recorded in terms of switches under off stimulation (*r* = 0.04, p = 0.87). Finally, we tested this result against the null hypothesis and we found a Bayes factor of 6.7, implying “positive” evidence (75–95%) in favour of the null hypothesis^36^.

### Simulated selections: the two functions of GPe

**Figure 4:**
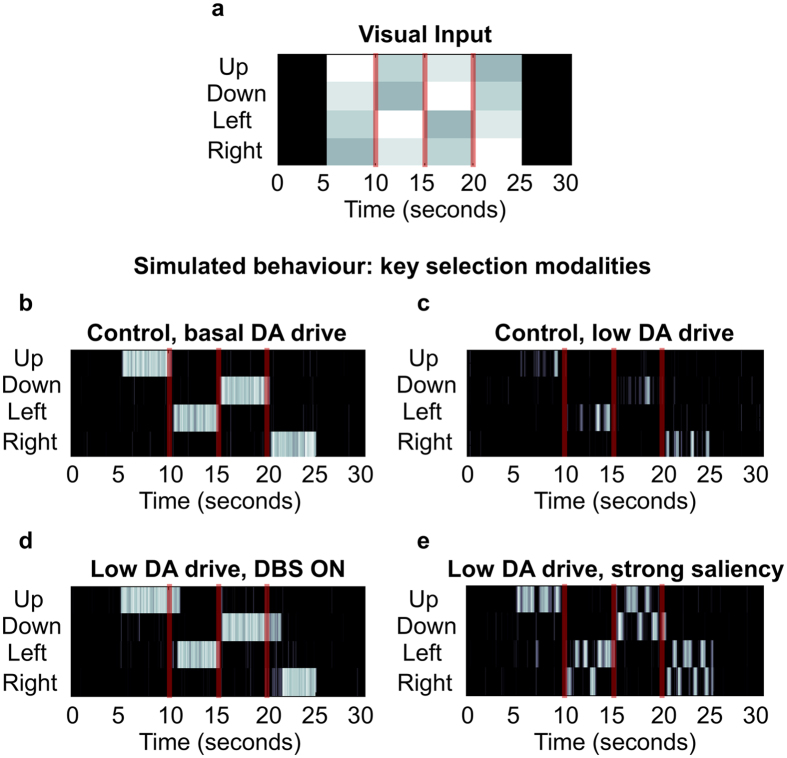
Simulated selections. An arbitrary visual input (**a**) representing four images characterised by different brightness, that change luminance pattern every 5 seconds. The first control simulation (**b**) shows the ideal behaviour: the artificial agent chooses in each interval the position associated to the brightest image (in this example, the correct sequence is: up, left, down, right). The condition of control is characterised by a set of parameters allowing quick initiation of selections as a response to the sensory input and continuous maintenance for the duration of the luminance pattern. The same parameters as the control, but for the reduced DA drive have been used to show the agent exhibits distinct behaviours, depending on the strength of the sensory stimulus. (**c**) Weak stimuli result in motor suppression; (**e**) conversely, strong sensory stimuli are associated with selections continuously changed with ambitendency among the strongest competitors. In both cases, the agent shows short perseverance after each change of luminance pattern. Finally, the simulated selections associated with Parkinson’s disease treated with subthalamic DBS (**d**) show the agent is again capable of performing correct choices and maintain a chosen selection. On the downside, the perseverance increases significantly as the agent shows problems in disengaging after a change in luminance pattern.

To account for the observed mismatches with the standard model, we explored the space of parameters offered by the connectome of the BG. We asked whether alteration of the effective connectivity resulting from sets of parameter configurations could generate variability in terms of motor selections under low dopaminergic drive. In the simulations, a visual input representing a luminance pattern of four different images (Fig. 4a) is encoded in the cortex with activity proportional to the perceived brightness. In a similar way, the simulated activity in the cortex encodes the saliency ascribed to the four cues in terms of strength of activation, so that high saliency is represented in the model by strong neural activity. On reaching the striatum, this pattern of activity is propagated as inhibitions towards GPi and GPe, thus preserving the encoded information about visual features and value, or saliency. This means the highest saliency in the cortex reaches both the GPi and GPe as the strongest inhibition. Finally the GPe propagates its activity pattern towards both STN and GPi via parallel inhibitions so that the GPi receives, with slightly different timing, undifferentiated activity from the STN, a saliency-specific pattern from the direct pathway and the opposite pattern from the short indirect pathway. If the balance between direct, hyperdirect and indirect pathway favours the first, the gain of the striato-cortical loop is strengthened, the network exhibits functional attractors and the behavioural output of the agent is input-driven maintenance, generally resulting in adaptive behaviours (Fig. 4b,d). By contrast, our investigation shows low dopaminergic drive results in more complex dynamics in terms of the effect it has on the attractor states of the network. Weakly valued stimuli reaching the striatum coupled with weak effective connectivity between GPe and the output nuclei of the BG favour the homeostatic loop established between hyperdirect and long indirect pathways (via STN). This activity results in the tonic excitation of the whole GPi, due to undifferentiated STN-GPi connections, therefore weakening the gain in the striato-thalamo-cortical loop, reducing attractor strength and causing motor suppression (Fig. 4c).

Conversely, low dopaminergic drive coupled with strongly valued stimuli reaching the striatum and strong effective connectivity from GPe to GPi, favours the short indirect pathway. Under this condition the short indirect pathway preserves the saliency values in its triple inhibition passages (Striatum-D2, GPe and GPi) making the striato-thalamo-cortical loop behave like a pattern generator, causing oscillatory activity among the channels and therefore switches of selections in presence of unaltered visual input (Fig. 4e).

The simulated case study illustrates how DA loss can result in selections being strongly influenced by GPe and the short indirect pathway, causing continuous change of selected motor activity, and consequently behavioural switching with a frequency in the range of around 0.5–2 Hz (Fig. 4e). This continuous switching among selected channels generates oscillations in the whole striato-thalamo-cortical loop which are significantly different from those postulated as caused by disruption of the homeostatic loop involving GPe and STN^11^. In the first case, the agent shows ambitendency, switching between different options and unable to preserve a selection. In the second case, the agent will continuously initiate and halt the same selected movement, showing repetitions (Fig. 4c, fourth interval: 20–25 seconds).

### Simulated selections: the mechanisms underlying subthalamic DBS

**Figure 5:**
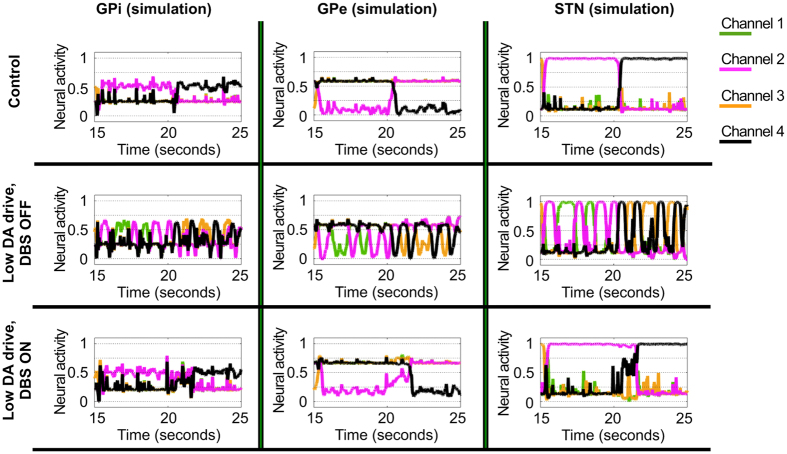
Simulated oscillatory neural activity in the BG under the condition of healthy control, low DA drive (DBS off) and high DA drive (DBS on). Simulated activity recorded in the Globus Pallidus (pars interna: GPi, and pars externa: GPe) and the subthalamic nucleus (STN) in the three conditions of control and low DA drive, on and off-stimulation. The graphs report 10 seconds of activity (range between 15 and 25) resulting in the action selection reported in the case study represented in Fig. 4. The colorcode is used to represent the activity of neural units belonging to the same channel, within different neural regions. The selected time interval highlights the presence of both slow (frequency of 0.5–2 Hz) and ultra-slow oscillatory patterns (frequency <0.5 Hz). The first type is mainly induced by the switching function realised by the short indirect pathway, whereas the second results from the maintenance function regulated by the direct pathway.

The above outlined new perspective about the role of the GPe allows simulating the effects of DBS by increasing the basal activity in the STN and both parts of GP (b_j_ in equation 1). Increased activity in STN provides a plausible explanation for increased Glu release in the GP, whereas increased activity in the GPe is known to be responsible for an increased GABA release in the GPi^20^,^23^. Off-stimulation, the simulations show the presence of slow (0.5–2 Hz) and ultraslow (<0.5 Hz) oscillatory activity in the STN and GPi in particular (Fig. 5: Low DA drive, DBS OFF), induced by the switching function (via GPe and short indirect pathway) and by the maintenance function (via direct pathway), respectively. On-stimulation, the ratio between signal and baseline activity changes in the whole GP favouring the latter, negatively affecting an ability of GPe to convey information about relative saliency of competing stimuli, thereby diminishing switching functionality and strengthening the functional attractor state the system has fallen into. The neural correlate for this alteration is represented as a decrease of slow oscillatory activity and enhancement of ultraslow oscillatory activity (Fig. 5: Low DA drive, DBS ON), in a way that resembles the effects caused by increased DA release^37^. Behaviourally, this alteration allows the system to mimic healthy selections but, on the downside, the loss of signal leads to increased perseverance (Fig. 4d), as we observed in the patients (experiment 1, see Fig. 3b). Interestingly, the simulations are also consistent with electrophysiology data^21^, reporting a majority of units downstream the locus of the stimulation show increased activation and others characterised by either decreased or unaltered activity (Fig. 5).

**Figure 6:**
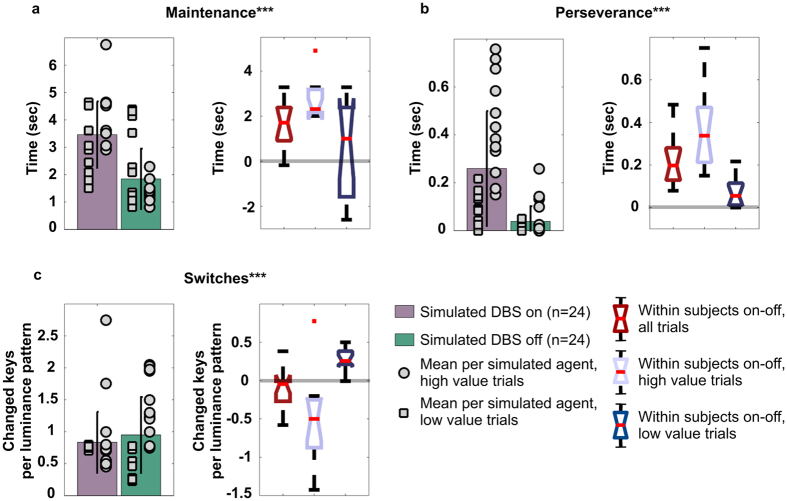
Action selections under on- and off- simulated subthalamic DBS. Indices of simulated behaviour: (**a**) maintenance, (**b**) perseverance and (**c**) switches of 12 randomly selected seeds, tested under four different conditions (on and off simulated treatment times high and low valued inputs). Histograms represent mean measures and standard deviation, per trial, under simulated on- and off- subthalamic stimulation, across reward conditions. Markers represent mean values per participant. Within each histogram, the markers have been grouped so that the squares represent measures recorded under the condition of low values and circles represent measures recorded under the condition of high values. Boxplots represent the distribution of within seed differences, with red markers used to highlight the presence of any outlier. Each measure reports the distribution of difference across value condition (left), and under either high (centre) or low (right) value separately. Both for the index of maintenance (**a**) and for the index of perseverance (**b**), the distribution of within seed differences shows these measures vary as a function of the treatment condition but they are not significantly affected by the value condition (F = 23.281, p = 0.001, and F = 39.881, p < 0.001, respectively). Conversely, in the measure of switches (**c**) we observed an interaction effect of the two variables of value and simulated treatment (F = 17.696, p = 0.001). When comparing on and off simulated DBS the number of switches decreases (standard motor suppression) under low values and it increases (excessive switching) under high value condition ((t(11) = 5.61, p < 0.001 and t(11) = −3.12, p = 0.009, respectively). If considered across value condition, the behaviour does not vary significantly as a function of the simulated treatment alone, which might explain the absence of effect found in the same measure for the first experiment, where no reward manipulation was included. Statistical significance (two-way repeated-measures ANOVA) expressed as follows: ***for p ≤ 0.001. Note we do not establish a quantitative comparison between the simulated selections and the reported behaviour of the patients as we prefer to highlight the direction of measure comparison. This choice is reflected in the simulation by the presence of slightly different timing and number of time intervals for the input changes.

The model predicts that, for a significant increase in switching function across subjects, two conditions must be met: the effect of DA on the indirect pathway has to be reduced (as in the case of Parkinson’s disease) and the activity pattern in the GPe has to encode strong saliency to trigger the motor oscillations. In our first experiment we do not control the value or saliency of the input. Therefore, to simulate the behavioural data, we have run a series of forty-eight simulations using twelve random seeds to control the cortical noise (see Methods section for details). Each of the twelve seeds represents a subject and has been tested four times, considering two sets of sensory inputs (high vs low values) combined with the two conditions of simulated subthalamic DBS activation (on vs off). The selections recorded under on- and off- simulated subthalamic stimulation successfully replicate the results described in the first experiment (cf. Figs 3a,c and 6a,c). Two-way repeated measures ANOVA reveals a main effect of the simulated DBS on the indexes of maintenance (F = 23.281, p = 0.001) and perseverance (F = 39.881, p < 0.001). The analysis of the measure of switches reports an effect of interaction between DBS condition and value associated with the input (F = 17.696, p = 0.001). Subsequent post hoc analysis replicates the target data. The agents under simulated treatment increase their chances to maintain a selection and persevere (t(23) = 3.94, p < 0.001, Fig. 6a; and t(23) = 5.21, p < 0.001, Fig. 6b, respectively), whereas the index of switches fails to show a significant variation as a function of treament (t(23) = 1.46, p = 0.16, Fig. 6c). Finally, the two hypothesised functions of motor suppression and motor switching are decoupled and clearly emerge as a function of both simulated treatment and input values (t(11) = 5.61, p < 0.001 and t(11) = −3.12, p = 0.009, when comparing on- and off- stimulation under low and high value conditions, respectively; Fig. 6c).

### Experiment 2: action selection under pharmacological manipulation

To validate our model, we aimed to replicate those conditions predicted to lead to significant increase in the number of actions in terms of switching, under low DA drive.

As was the case in our first experiment, participants were required to select the brightest image among four squared tessellation figures in grey scale (Fig. 2). Two levels of reward associated with each trial (either £1 or £10) were introduced to comply with the required presence of cues associated with differential saliency. Finally, we introduced a pharmacological manipulation (see methods for details) to allow comparison between placebo and either low or high DA drive conditions. By this, we aimed to establish a comparison with data collected in Parkinson’s disease patients (low DA drive, induced administering DA antagonist) and, as a secondary goal, to explore potential interaction effects across a wider spectrum of altered DA drive (specifically, enhanced DA drive, induced administering DA precursor).

Possibly due to the small number of participants in the experiment (17 valid participants), the analysis of the behaviour in this study is inconclusive. Two-way repeated measures ANOVA reports no significant effect in the measure of maintenance and perseverance, with only a weak trend in the interaction between pharmacological manipulation and reward on the measure of switches (F = 2.501, p = 0.098).

**Figure 7:**
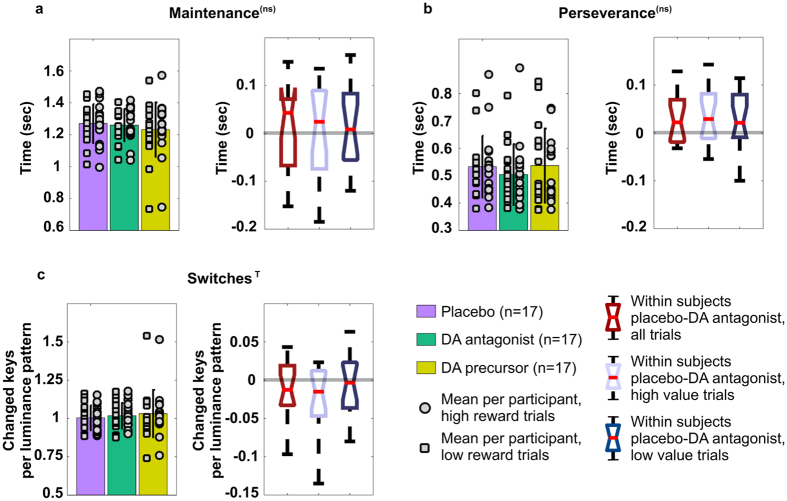
Behavioural indices recorded in Experiment 2. (**a**) maintenance, (**b**) perseverance and (**c**) switches, in 17 healthy participants under three conditions of pharmacological manipulation. Histograms represent mean values and standard deviation under placebo, DA antagonist (risperidone), and DA precursor (L-Dopa). Markers represent mean values per participant, grouped so to represent measures recorded under the condition of either low or high values (squares and circles, respectively). Boxplots represent the distribution in quartiles of within subject differences between measures recorded under placebo and DA antagonist: the two conditions have been selected to establish a comparison with experiment 1. Each measure reports the distribution of differences across value condition (left), and under either high (centre) or low (right) value separately. Two-way repeated measures 3 × 2 ANOVA indicates the presence of a trend in the interaction between pharmacological and reward conditions for the measure of switches (F = 2.501, p = 0.098). Follow-up analysis reveals a main effect of the pharmacological manipulation (F = 5.296, p = 0.035), irrespective of the associated reward, when comparing the measure of perseverance recorded under placebo and DA antagonist condition (**b**, right). Finally, two-way repeated measures 2 × 2 ANOVA confirms the presence of an interaction effect for the index of switches when comparing behaviour under DA antagonist with either placebo (**c**) or DA precursor (F = 3.121, p = 0.096 and F = 6.492, p = 0.021). In particular, the distribution of within subject differences (Placebo -DA antagonist, **c**, right), recorded under high reward condition, shows the predicted increase in terms of number of switches per luminance pattern under the low DA drive pharmacological manipulation. Statistical significance in the figure, relative to the preliminary 3 × 2 ANOVA is expressed as follows: ^T^ for 0.10 > p > 0.05; ^(ns)^ for not significant.

For exploratory purposes, we ran follow-up 2 × 2 repeated measures ANOVA for each dependent variable of interest. This analysis showed a main effect (F = 5.296, p = 0.035) of pharmacological manipulation on the measure of perseverance when comparing placebo vs DA antagonist. T test confirmed the behaviour recorded under these conditions is consistent with data collected in the first experiment and the predictions of the model. Thus as predicted, the measure of perseverance shows an improved ability to disengage under DA antagonist, irrespective of reward condition (placebo: 533.13 ms vs DA antagonist: 504.22 ms, t(16) = 2.30, p = 0.035, Fig. 7b).

For the measure of switches, 2 × 2 ANOVA confirms an interaction between pharmacological manipulation and reward, when comparing behaviour recorded under either placebo or DA precursor with behaviour recorded under DA antagonist (F = 3.121, p = 0.096 and F = 6.492, p = 0.021, respectively). Finally, and in keeping with predictions of the model, we observed a significant increase in the number of switches under DA antagonist vs placebo in high reward condition (placebo:6.07 vs DA antagonist:6.14, t(16) = −2.17, p = 0.045, cf Fig. 7c). On this measure it should be noted that we were expecting to record the standard effect of motor suppression under low DA drive condition coupled with low value, which is not reported (p = 0.60). We hypothesise that the applied low reward (£1) manipulation may still have been considered as somewhat salient by the participants. In a subjective state questionnaire the participants estimated, in a scale one to five, their own motivation in dealing with the different trials. The mean difference in these self-evaluations is equal to 1.04 (SE = 0.13) in favour of high reward trials.

## Discussion

The basal ganglia (BG) form key neuronal circuitry for action control whose dysfunction is involved in a large number of brain disorders. Understanding how this circuitry regulates action selection, initiation and maintenance under aminergic neuromodulation can elucidate pathophysiological commonalities and differences between brain disorders and thus guide therapy. According to the standard model, action control critically depends on competing D1/D2 pathways that facilitate and suppress motor output. While this model has set a standard since the 1980s, it fails to explain recent clinical and experimental data. For example, varying therapeutic efficacy between movement disorders with overlapping clinical phenotypes, as seen in Parkinson’s disease and atypical Parkinson syndromes, is not accounted for in this model.

The standard model was developed to explain the mechanisms underlying behaviour recorded in experiments requiring selection of actions on individual trials. In contrast, here we test adaptive sensory-motor responses to a changing environment and maintenance of an action until it is no longer suitable. The visual discrimination task we propose allows examining how motor flexibility is altered under low DA drive, as in Parkinson’s disease. We rely on three indices to measure motor flexibility, termed maintenance, perseverance and switches. We show that reduced DA outflow off subthalamic stimulation, compared with on-stimulation, results in a reduced ability to maintain a selection, whereas on-stimulation behaviour is characterised by prolonged perseverance, or time for disengaging (Fig. 3). Statistical correction for multiple comparisons, such as Bonferroni’s alpha level of 0.016 for the three indices, would make only the first of these comparisons statistically significant. Nonetheless it should be noted that this correction can be too conservative due to the high correlation among the indices (sup. Fig. 1). Our data suggest reduced DA drive is not necessarily correlated with reduced motor activity, as postulated in the classical positive correlation between DA release and motivation or vigour^38^,^39^. The simulations in the proposed neural model of the BG show it is possible to generate motor changes in selection performed when the input remains unaltered and motivation or vigour is predicted to be reduced due to attenuated DA drive. In the model, this type of dysfunctional switching is caused by the triple inhibitions characterising the short indirect pathway and oscillatory patterns are triggered by the information encoded in the GPe and propagated towards the GPi via direct inhibitions.

On this basis we predict an increase of unnecessary motor switching is associated with activity in the indirect pathway, in presence of reduced DA drive and highly valued stimuli, as illustrated by the model simulations. The task designed for the first experiment was associated with a generic compensation for the time spent performing the task, but there was no specific condition characterised by either high or low rewards, so that the experiment could not evoke a significant increase in switching under the off stimulation condition. In our second experiment we aimed to investigate the interactions between a pharmacological manipulation (either DA precursor or blockade of DA receptors) and stimuli associated with different saliency (high and low rewards). The dopaminergic blockade and the placebo conditions were included to establish a comparison with Parkinson’s disease patients under off-on- subthalamic stimulation, respectively. The inclusion of the DA precursor condition was meant to highlight the non-linearity of the interaction between value and dopaminergic drive. The results of this second study were inconclusive, showing only a trend effect which was nonetheless in keeping with the predictions of the revised model. Follow-up tests were also consistent with both data collected in the first experiment and the proposed model and if confirmed in further studies, would pose an important challenge to the standard concept of the indirect pathway as only controlling motor suppression.

The new framework we propose exploits the functional connectivity among GPe and either GPi (in the sensorimotor loop) or the Substantia Nigra pars reticulata (SNr, in the ventral loop) which we suggest has been so far neglected, limiting the predictive power of the standard rate model. These efferent connections from the GPe towards its targets in the output nuclei of the BG have been used in our model to generate excessive switching via concurrent activity of direct and short indirect pathway, forcing the agent to change selections in a way that resembles a weak ambitendency. Significantly, this same circuit is found in controlling selections and coupling different parts of the striatum and the cortex. For instance, increased switching induced via GPe-SNr connections in the limbic striato-cortical loop (involving prefrontal cortex and ventral striatum) may be revealing for apparently conflicting phenomena like ADHD, where hyperactivity is effectively treated by increasing the dopaminergic drive^40^.

**Figure 8:**
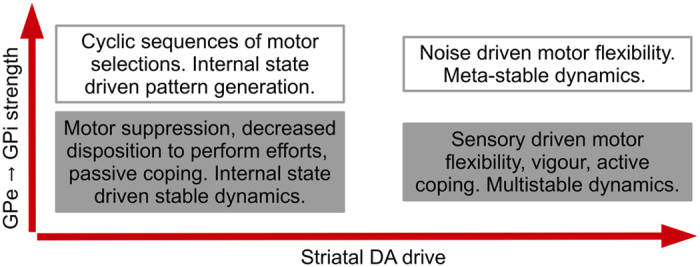
Diagram of motor functions expressed by the standard and the enriched model. Four motor modalities are here represented as ideally separate sets for illustrative purposes. The standard framework mainly includes the motor modalities associated with vigour or motivation (grey boxes). These motor functions can be spatially represented on a single axis, as a function of striatal DA drive. Conversely, the proposed enriched model includes all four modalities (grey and white boxes), and requires a spatial representation on two axes, as a function of both striatal DA drive and strength of information propagated via the short indirect pathway (i.e. via direct inhibitions from GPe to GPi). The diagram illustrates the more comprehensive representation offered by the enriched model does not exclude any of the modalities presented in the standard model, and at the same time it allows a more precise association of each modality with specific neural causes.

The recent availability of more sophisticated ways to manipulate the BG circuitry and record its activity indicates that the standard model does not account for a wide range of human and animal data^2^. Optogenetic studies show results incompatible with a mutually exclusive facilitative and suppressive pathway architecture, and highlight the co-occurrence of activity within both pathways when motor activity is generated^41^,^42^. Our investigation points out that activity in the indirect pathway is consistent both with the function of regulating motor suppression via STN^9^,^10^,^11^ as well as with the function of generating oscillatory selections or motor switching in general (via internal connectivity in the GP). It is due to this enriched view (Fig. 8) that we argue it is possible to reconcile the apparently conflicting findings regarding a concurrent role for the indirect pathway in promoting action^43^ and the known strong correlation between reduced dopaminergic drive and motor suppression^38^. Indeed the model predicts that even in healthy subjects, at basal DA release, the same mechanism can be exploited to make use of the BG as a pattern generator. Cortical-striatal learning processes weigh saliency in the striatum, so that they can potentially increase the value encoded in the sensory input^44^. It is then possible for the system to associate a sensory input to cyclical selections of motor responses, enabling contraversive movements requiring a cyclical frequency of activation as, for instance, walking^45^.

Activity in the GPe affects the gating system of GPi in two ways, controlling general basal activity (via STN) and encoded signal (via direct inhibitions). The overlap of these two different effects is essential to explain the elusive therapeutic mechanism of DBS. The simulations show that, by increasing activity of both STN and GP, subthalamic DBS can alter the ratio between signal and basal activity, leading to diminution in relative strength of information conveyed by the GPe in the indirect pathway. This alteration accounts in the simulations for a diminished switching functionality in the BG, which results in higher probability to maintain an action selection for the time required to perform the selected action, so offsetting motor disabilities caused by Parkinson’s disease. On the downside, this reduced ability to switch makes it more likely for the patients to ignore changes in the environment and maintain a selected action, increasing perseverance, as observed in the patients in the on-stimulation condition. We speculate that, if this same mechanism is associated with activity in a limbic striato-cortical loop (which might be affected by the presence of the stimulator in the STN), it can lead to obsessive compulsive like behaviour.

Finally, the use of rate neurons in the presented model does not account for responses recorded in association with DBS treatments in the order of milliseconds^31^,^46^. Instead we aimed to simulate average effects recorded over a timescale of seconds and their emergence from the systemic interaction between the neural nuclei involved. The global activity of the BG is characterised by a wide variety of oscillatory patterns both in healthy and in pathological conditions^47^,^48^ and neither their cause nor their relation with expressed behaviour is fully understood. Oscillations have been recorded in rat and monkey models of Parkinsonism^37^, described as either slow (0.3–2 Hz) or ultraslow oscillations (0.017–0.5 Hz). Both patterns are affected by DA release, with slow oscillations enhanced by DA depletion and ultraslow oscillations enhanced by increased DA stimulation^37^. Slow and ultraslow field potentials oscillations in the BG are characterised by dynamics which are very similar to our observed simulated oscillatory patterns. Action selection in the model is represented by changes in activity in the channels of the simulated BG. Each of the four channels we have designed for the artificial neural circuit is uniquely associated with one of the four action selections allowed by the task. Recent findings support the presence of such localistic representation in the BG^49^, allowing a loose comparison between simulated data and real behaviour when comparing oscillations. Long phase change in the model activity are determined by the architecture of the neural network, therefore, the model is reasonably accurate in describing causal dynamics leading to slow and ultraslow oscillations. Decreased dopamine in the striatum leads to decreased gain in a striato-thalamo-cortical loop and as a consequence lower stability of the system, with faster changes of activity among channels and oscillations. This implies direct pathway activity can be associated with ultraslow oscillations and indirect pathway activity with slow oscillatory patterns.

Concerning slow oscillatory patterns, we propose the GPe-STN homeostatic loop and the GPe-GPi connectivity are the generators of at least two different types of patterns, with the model highlighting their structural and (consequently) qualitative differences. Different equilibrium states among these nuclei can account for inter-patient behavioural variability which may be expressed as distinct clinical phenotypes. In particular, the model implies that, since motor modalities can be expressed as a function of both striatal DA drive and GP internal connectivity, inter-subjective difference in the internal connectivity of the GP can be a potential neural correlate for two opposite behaviours expressed in association with DA loss in the striatum, namely hypokinesia and hyperkinesia. The variability in the repertoire of actions recorded in association with reduced DA outflow is a putative signal of such phenotypes (Fig. 8). Further model development is required, including a more complex representation of the organization of the striatum, and the inclusion of a more realistic catecholamine release simulation as part of a further homeostatic loop. Such improvements may then help simulate differences in clinical phenotypes and treatment response across patients with overlapping symptoms (e.g., Parkinson’s disease and atypical Parkinson syndromes), as well as for phenotypic overlap between classically distinct BG disorders (e.g. Parkinson’s and Huntington’s). This clarification of distinct versus overlapping pathophysiological principles across movement disorders has the potential to refine future clinical practice.

## Methods

### Participants

The first study included twenty-one patients with Parkinson’s disease (15 male, age range: 40–73 years; 19 right handed) who had been selected on clinical grounds for treatment using bilateral DBS of the STN. Patients were recruited from the Departments of Neurology and Stereotactic Neurosurgery at the University of Magdeburg and the diagnosis of Parkinson’s disease was confirmed by a neurologist specialized in movement disorders. The mean duration since DBS surgery was 44.3 ± 28.8 months. Demographic and disease characteristics of each patient can be seen in Table 1. All patients remained on their prescribed dopaminergic medication in conjunction with DBS and were tested during their normal medication cycle. All patients had opted for DBS surgery because their medications were either causing side effects or were no longer providing optimal control over motor symptoms.

The surgical procedure for STN DBS utilized standard stereotactic techniques with microelectrode recordings for electrophysiological localization^50^. Electrodes were placed bilaterally in the STN of all patients. Briefly, macroelectrodes (Medtronic Model 3389) consisting of four platinum–iridium cylindrical surfaces, each with a diameter of 1.27 mm, length of 1.5 mm and edge-to-edge separation of 0.5 mm, were guided into the STN using MRI-guided stereotaxy and intraoperative microelectrode recordings. The planned coordinates for macroelectrode placement were based on direct visualization of the STN on T2-weighted magnetic resonance images. Final electrode position was based on microelectrode recordings and confirmed intraoperatively with macrostimulation after implantation of the DBS electrodes. Selection of final bipolar contacts and stimulation settings were determined on an individual basis to optimize control over clinically manifest motor symptoms.

All patients were free of dementia and did not show clinical levels of depression at the time of testing. Further exclusion criteria included a history of neurological condition other than Parkinson’s disease, any psychiatric condition known to compromise executive cognitive functioning (e.g. schizophrenia, bipolar affective disorder, mood disorders) or any untreated or unstable medical conditions. All patients participated voluntarily and could quit the study at any time. The experiment was approved by the local ethics committee (University of Magdeburg, Germany) and informed consent was obtained from all participants.

The task reported here was part of a battery of four separate tasks, each lasting roughly 15 minutes. All four tasks were repeated twice, on- and off-stimulation, with a break of at least one hour between the two sessions, resulting in a total of three hours for the whole testing. Session order was counterbalanced across subjects. 12 patients completed the task starting with the on condition and 9 completed the task starting with the off condition. Of the twenty-five patients initially included in the study, three did not complete the task in both conditions (due to tiredness), and one patient made only one selection (out of the six required) in almost half of all trials both on and off-stimulation. These patients were therefore excluded from analysis.

The second study included seventeen healthy volunteers (8 male, age range: 18–35 years; all right handed; weight range: 50–70 kg) who responded to a call for participants distributed in a mailing list by the Institute of Cognitive Neuroscience (UCL, London). Exclusion criteria included any history of mental disorder or drug abuse. Participants were also required to avoid consumption of alcohol, coffee or tea (or any similar stimulant usually present in a common energy drink) within at least 12 hours before each day of testing. All participants were aware that they could quit the study at any time. The experiment was approved by the local ethics committee (University College London) and informed consent was obtained from all subjects. They were asked to attend the Functional Imaging Laboratory three times, one per pharmacological condition, on a weekly basis with the condition order counterbalanced for all subjects. The schedule consisted in administering a first juice one hour prior the task, for the placebo (vitamin C) or DA antagonist (0.5 mg risperidone), and a second juice 30 minutes prior the task, for the placebo or DA precursor (150 mg L-dopa). Vitamin C tablets were used as placebo to mimic the remains of L-Dopa tablets at bottom of the glass. An authorised medical doctor administered the drugs and was present during the whole time of the study. This schedule was conceived to maximize the effect of both drugs, preserving the requirement of a double blind investigation. The training of the participant was conducted after the administration of the second juice in all conditions and it was repeated until the subject reached an accuracy of at least 50% on average.

Of the eighteen subjects initially included in the study, data from seventeen were used for the analysis. One volunteer was excluded from the analysis due to excess alcohol consumption in the twelve hours prior to one of the sessions, which led to a poor performance. The participant was marked as an outlier both in terms of overall accuracy (percentage of time spent selecting the correct key: 35.4% vs an average of 46.78%) and in terms of time spent without performing any selection (seconds of inactivity per trial: 3.18 vs an average of 1.58). In the described T tests comparisons under the key conditions of placebo and DA antagonist, the inclusion of the outlier reduces the significant difference found in both the measure of perseverance across reward condition (t(17) = −1.79, p = 0.09) and the measure of switches under high reward (t(17) = −1.86, p = 0.079). Finally, the only trend reported in the two-way repeated-measures 3 × 2 ANOVA would not be present, if the outlier were to be included in the analysis.

All experiments were performed in accordance with relevant European and national guidelines and regulations.

### Task and behavioural indices

The goal of the task was to probe patterns of action selection by dissociating action maintenance, defined as the length of time intervals spent pressing any keyboard key, correct or not, irrespective of whether the luminance pattern had changed before or not; perseverance defined as the time required to disengage from any selected key, after each change in the stimulus; and switches, where there are changes of selections performed while the stimulus is constant, such that a new selection is not caused by a change in perceived luminance pattern.

Each trial started with four images appearing on the monitor after 2 seconds of a blank screen showing only the fixation cross (Fig. 2). Next, the luminance pattern changed five times during each trial of experiment 1 and six times during each trial of experiment 2. Changes occurred with a variable interval of 2–4 seconds (experiment 1) and 1.25–2 seconds (experiment 2), resulting in an overall trial length of 15–20 seconds (experiment 1) and 10 seconds (experiment 2). Importantly, the pace with which the environment changed was unpredictable and the timing in the two experiment was significantly different to adjust to the time required by the different subject pools to respond to the sensory stimuli. This pace was conceived so to avoid known effects of low DA on effort-related selection^38^ and learning^51^, circumventing complicated interpretation of behavioural measures. Participants were asked to press the key, among the four available arrow keys of a standard computer keyboard, corresponding to the position of the brightest image on the screen. Once a key was pressed, a grey line appeared close to the selected image and disappeared if more than one key was pressed at the same time. In the first experiment, subjects were presented with 3 blocks of 8 trials each, with a fixed interval of 3 seconds to separate the trials and a break at the end of each block. In the second experiment, subjects were presented with 12 blocks of 8 trials each, with a fixed interval of 6 seconds (including 3 seconds showing a label for the monetary reward) to separate the trials and a break at the end of each block.

Participants were fully instructed and completed at least one full training block before starting the actual experimental task in each condition of stimulation or pharmacological manipulation. No specific strategy was suggested and either the right or left hand could be used to emit a response, as patients and healthy volunteers felt more comfortable.

To motivate participants in experiment 1, the task was presented as a game and a score was provided at the end of both training and actual sessions, representing the percentage of time spent selecting the correct figure. This index of accuracy was computed by dividing each trial into intervals of 17 ms circa (the finest grain allowed by the software) and by comparing for each interval the selection performed (if any) and the actual brightest cue on the monitor. A perfect match would have resulted in a score of 100. The same index was used for the healthy volunteers but in this case a reward of either £1 or £10, each in 50% of trials, was pseudo-randomly associated with each trial. Thus, in the second experiment, the earned reward was proportional to the achieved accuracy score and each block of 8 trials offered a total of £44 for those able to perform with 100% of accuracy (4 trials worth £1 plus 4 trials worth £10). A label at the beginning of each trial and prior to the fixation point was used to indicate the reward associated with the upcoming trial. At the end of each experimental condition, one block of trials was randomly selected and the participant nominally received the corresponding reward. All rewards were actually paid out after completion of all experimental conditions, i.e., after the last of the three weekly sessions. The goal of the participants was to maximise their score in the first experiment and maximise the reward in second one.

We were interested in three behavioural indices: firstly, a maintenance index, reflecting the time spent keeping a key pressed, independently of the visual input. Secondly, a perseverance index, reflecting the time the participants required to realise the a luminance pattern has changed and release a current selection (if any). Note that responses that were faster than 300 ms (experiment 1) or 200 ms (experiment 2) were excluded from all analyses to avoid false positives provided by involuntary movements (hence the longer interval used for Parkinsonian patients^52^). Finally, we measured the number of times the participants changed their selections during a single luminance pattern. Each luminance pattern required a single selection or switch, but the participant could at any time change his or her mind and perform a new selection. We did not consider a “switch” any repeated selection of the same key (e.g. a key is released for any reason during a luminance pattern and then pressed again).

The hypothesis of null finding for this measure has been tested relying on a Bayesian method^53^, predicting a non-uniform distribution (mean = 0, standard deviation = 0.2 and tails = 1), and considering the prediction that reduced DA drive is highly correlated with reduced motor activity and flexibility.

### Computational Model

The model focusses on the dynamics of neural substrates and is constrained by known neuroanatomical data regarding the connectivity of the entire BG. The model encompasses elements of existing computational models of the BG^2^,^54^, including neural models relying on either spiking or rate units, developed to exploit a microconnectivity creating functional macro and micro “channels” within the BG^4^. Three segregated re-entrant striato-thalamo-cortical loops have been described in humans, involving motor, associative and limbic areas^55^ where each loop replicates the same intrinsic structure, being divided into several channels and processing its inputs in parallel. The somatotopic organisation in motor cortex is generally preserved throughout the motor loop and is found within striato-pallidal pathways, STN as well as thalamus^12^.

Our model simulates the activity of four channels in the motor loop. For the present rate neural network, we further developed C++ libraries^32^,^34^ to comply with the new requirements. In this new version, the neural architecture has been significantly altered to include a thalamo-cortical re-entrant loop as well as the indirect pathway. The average activity of an entire pool of neurons is simulated via a leaky integrator, where the action potential is described by equation (1), and equation (2) describes the transfer function determining the final activation of the unit:


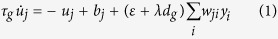



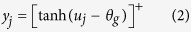


where τ_g_ represents a time constant related to the nucleus g to which unit j belongs, u_j_ is the activation potential of unit j, b_j_ is the basal activation of such unit, w_ji_ represents the connection weight between input unit i and unit j, and y_i_ the activation of input unit i. For the cortical layers, differentiated noise has been introduced in each unit via random alteration of the value of b_j_ in the interval ± 0.1. Noise time series used in the simulation are controlled by specific “seeds” so to enable repeated simulations (i.e. different conditions) with the same noise. In the analysis of the simulated selections, the twelve seeds were controlled so to run with each of them four simulations under different conditions (two by two: on or off simulated subthalamic DBS and high or low valued inputs). This procedure is required to enable a comparison of the final output of the system, so that the reported differences can be considered as caused only by the variation established in terms of simulated conditions. The amount of DA release reaching the group is represented by d_g_ and the coefficients ε and λ are set to 1 and 0 respectively for all units, except for the units in the striatum. In the striatum, they are set to ε = 0.2 and λ = 1 for D1 units and ε = 0.6 and λ = −0.5 for D2 units, to account for the differential effect DA has on the two populations of units in the striatum. The transfer function (equation 2) is characterised by an hyperbolic tangent tanh(.) of the action potential u_j_ minus a threshold θ_g_, which is the same for the whole group g. Note that θ_g_ > 0 only for cortical layers. The positive function [.]^+^ is defined as [x]^+^ = 0 if x ≤ 0 and [x]^+^ = x if x > 0. As a consequence, 0 < y_i_ < 1 for all j.

The frequency of the oscillations in the model is determined by both the neural architecture of the model, and by the time constant τ_g_ regulating the action potentials of the neural units as described in equation 1. The latter is the reason why the model cannot represent phase changes taking place with a frequency above 2 Hz. Therefore, the more the simulation gets close to this limit, the more it is limited by the presence of this time constant and its dynamics are less reliable. Conversely, longer phase changes are only determined by the architecture of the neural network.

The external visual input to the network is represented by a four dimensional vector, whose fixed values change disposition with a fixed interval of 5 seconds. The vector of values used to simulate conditions of high saliency is characterised by the following values: [0.5 0.45 0.4 0.35], whereas the condition of low saliency is represented by the following: [0.4 0.35 0.3 0.25]. The complete neural architecture used for all simulations is represented in supplementary Fig. 2.

The exploration of the space of parameters relied on hand-tuning and trial-and-error optimization. The parameters have been hand-tuned to find a configuration that allows for both increased and decreased motor activity under low dopaminergic drive, depending on an independent variable. The same values for the coefficients ε-D1 and λ-D1 have been used as described in previous work^34^. These are meant to allow small signal transmission between cortex and striatum in the case of null presence of dopamine. The values of ε-D2 and λ-D2 have been tested to maintain slightly greater signal transmission in the indirect pathway, in comparison with the direct pathway, under a condition of low dopaminergic value. The model is sensible to a variation in these parameters, but the effect on switching caused by the short indirect pathway (as discussed in the paper) can be preserved in any configuration allowing i. robust activity in the indirect pathway under low dopaminergic drive and ii. at the same time a balance among the three GPi afferents: D1-striatum, STN and GPe.

## Additional Information

**How to cite this article**: Fiore, V. G. *et al.* Changing pattern in the basal ganglia: motor switching under reduced dopaminergic drive. *Sci. Rep.* **6**, 23327; doi: 10.1038/srep23327 (2016).

## Supplementary Material

Supplementary Information

## Figures and Tables

**Figure 1:**
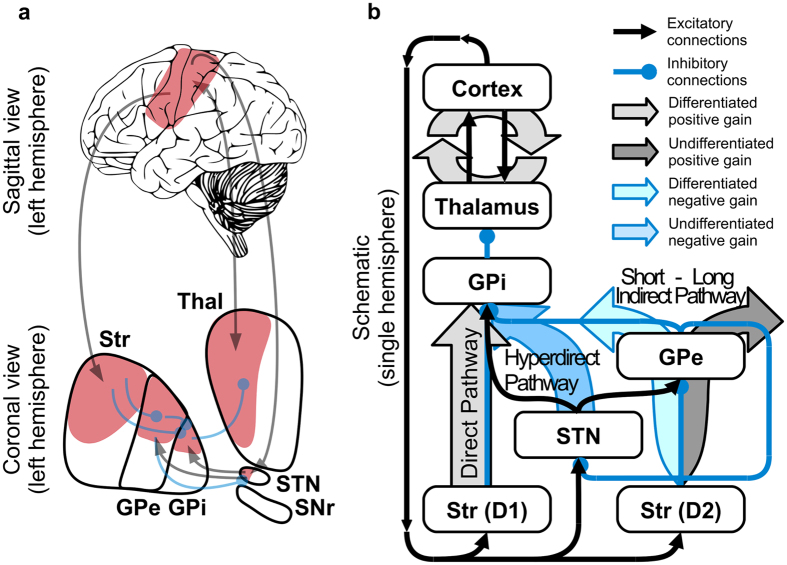
Direct, hyperdirect and indirect pathways of the basal ganglia. (**a**) Anatomical (left) and (**b**) schematic (right) representations of a sensorimotor striato-thalamo-cortical circuit highlighting the presence of the major pathways and the way they contribute to the gain in the neural circuit. Thal = thalamus; GPi = globus pallidus pars interna; GPe = globus pallidus pars externa; STN = subthalamic nucleus; SNr = Subtantia Nigra pars Reticulata. Str (D1) and (D2) represent the areas of the striatum characterised by high concentration of either D1 or D2 receptors.

**Table 1:** Parkinson’s disease patients and their treatment.

Patient #	Age [years]	Gender	Disease duration [years]	assessment post surgery [months]	UPDRS III Score On/Off	LED [mg/d]	DBS contacts	DBS voltage [V], frequency [Hz], pulse with [μs]
1	58	m	32	33	16/	100	0− 2+/8− 10+	2,5 V, 130 Hz, 60 μs/2, 7 V, 130 Hz, 60 μs
2	64	m	10	57	16/32	705	3− 1+/7− 5+	5, 0 V, 130 Hz, 60 μs/4, 5V, 130 Hz, 60 μs
3	67	m	17	69	8/	780	2− G+/4− 6+	2, 0 V, 130 Hz, 60 μs/3, 5 V, 130 Hz, 60 μs
4	65	m	14	13	17/21	0	2− G+/9− G+	4, 5 V, 130 Hz, 60 μs/3, 7 V, 130 Hz, 60 μs
5	67	f	34	120			C+6−/C+3−	2, 3 V, 180 Hz, 60 μs/2, 3 V, 180 Hz, 60 μs
6	63	f	23	36	12/33	920	9− G+/2− G+	2, 5 V, 130 Hz, 60 μs/3, 5 V, 130 Hz, 60 μs
7	56	m	9	9	2/21	0	2− G+/10− G+	2, 0 V, 130 Hz, 60 μs/2, 5V, 130 Hz, 60 μs
8	53	m	6	52	1/29	326	1− 2+/9− 11+	2, 0 V, 130 Hz, 60 μs/4, 0 V, 130 Hz, 60 μs
9	59	m	11	28	15/27	0	2− G+/11− G+	1, 1 V, 60 Hz, 60 μs/3, 6 V, 60 Hz, 60 μs
10	65	f	10	57	5/27	0	1− G+/6− G+	2, 0 V, 130 Hz, 60 μs/2, 5V, 130 Hz, 60 μs
11	40	m	5	12	22/26	0	3− G+/11− G+	2, 0 V, 130 Hz, 90 μs/2, 0 V, 130 Hz, 90 μs
12	58	m	12	50	8/38	402	1− G+/9− G+	2, 1 V, 160 Hz, 60 μs/2, 1 V, 160 Hz, 60 μs
13	68	m	12	12	/31	255	1− G+/9−G+	1, 4 V, 180 Hz, 60 μs/2, 8 V, 180 Hz, 60 μs;
14	54	m	14	49	9/28	310	3− G+/11− 10+	1, 6 V, 180 Hz, 60 μs/4, 5 V, 180 Hz, 60 μs
15	55	m	9	35	28/28	1400	C+3−/C+11−	2, 8 V, 130 Hz, 90 μs/2, 0 V, 130Hz, 90 μs
16	61	m	12	56	6/15	150	G+3−/G+11−	3, 0 V, 125 Hz, 60 μs/2, 6 V, 125 Hz, 60 μs
17	53	f	9	8	/26	0	0− G+/8− G+	2, 0 V, 130 Hz, 60 μs/2, 1 V, 130 Hz, 60 μs
18	67	m	8	55	16/24	1010	2− G+/10− G+	0, 8 V, 130 Hz, 60 μs/2, 4 V, 130 Hz, 60 μs
19	70	m	?	74	15/	500	3+2−/7+6−	4, 0 V, 130 Hz, 130 μs/4, 0 V, 130 Hz, 130 μs
20	62	f	20	18	/40	530	2− G+/10− G+	3, 5 V, 180 Hz, 60 μs/3, 5 V, 180Hz, 60 μs
21	70	f	17	87			1− G+/4− G+	3, 6 V, 180 Hz, 60 μs/4, 2 V, 180 Hz, 60 μs
