# Corrigendum: Changing pattern in the basal ganglia: motor switching under reduced dopaminergic drive

**DOI:** 10.1038/srep30841

**Published:** 2016-08-19

**Authors:** Vincenzo G. Fiore, Francesco Rigoli, Max-Philipp Stenner, Tino Zaehle, Frank Hirth, Hans-Jochen Heinze, Raymond J. Dolan

Scientific Reports 6: Article number: 2332710.1038/srep23327; published online: 03 2016 2016; updated: 08 19 2016.

This Article contains an error in the Introduction section.

“The standard model predicts that a normal balance between competing BG pathways can be restored by decreasing indirect and hyperdirect pathway activity, for example via lesions of the globus pallidus pars externa (GPe) or the STN.”

should read:

“The standard model predicts that a normal balance between competing BG pathways can be restored by decreasing indirect and hyperdirect pathway activity, for example via lesions of the STN.”

