# Supplementary material for: Changing pattern in the basal ganglia: motor switching under reduced dopaminergic drive
Source: Sci Rep. 2016 Mar 23;6:23327. doi: 10.1038/srep23327 (PMC4804216; doi:10.1038/srep23327)
Supplement: Supplementary Information [file srep23327-s1.pdf]

# Changing pattern in the basal ganglia: motor switching under reduced dopaminergic drive

Vincenzo G. Fiore[1*], Francesco Rigoli[1], Max-Philipp Stenner[1,2], Tino Zaehle[2], Frank Hirth[3], Hans-Jochen Heinze[2,4], Raymond J. Dolan[1,5]

[1] Wellcome Trust Centre for Neuroimaging, University College London, 12 Queen Square, London WC1N 3BG, United Kingdom

[2] Department of Neurology, Otto-von-Guericke-University Magdeburg, Leipziger Str. 44, 39120 Magdeburg, Germany

[3] King's College London, Institute of Psychiatry, Psychology & Neuroscience, Department of Basic & Clinical Neuroscience, London, UK

[4] Department of Behavioral Neurology, Leibniz Institute for Neurobiology, Brenneckestr. 6, 39118 Magdeburg, Germany

[5] Max Planck UCL Centre for Computational Psychiatry and Ageing Research, 10-12 Russell Square, London, WC1B 5EH, United Kingdom

*Corresponding author:

Wellcome Trust Centre for Neuroimaging at UCL

12 Queen Square - London - WC1N 3BG

Tel: +44 (0)20 3448 4362

email: v.fiore@ucl.ac.uk

**Running Title:** Changing pattern in the basal ganglia

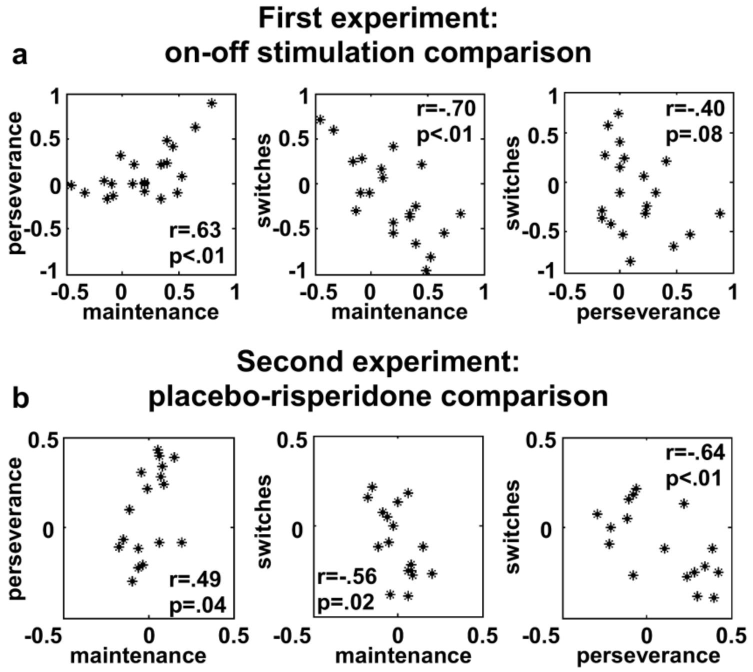


Supplementary figure 1 – **Correlations**. The scatterplots report the degree of correlation among the three indices used in the first (a) and the second (b) experiments.

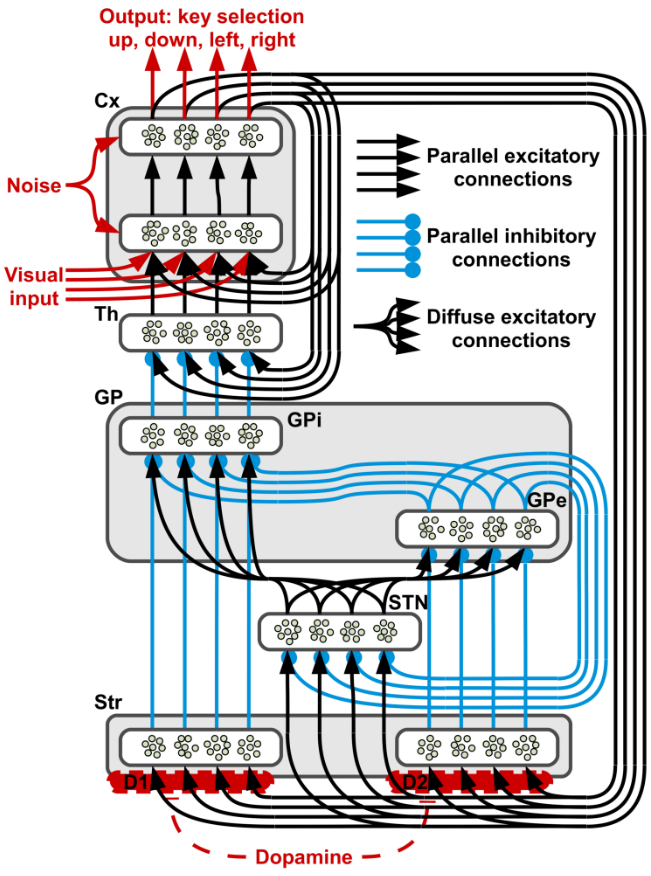


Supplementary Figure 2 – **Neural architecture used to simulate all the reported conditions.** Cx= cortex; Th= thalamus; GP= globus pallidus (GPi=pars interna, GPe= pars externa); STN= subthalamic nucleus; Str= striatum; D1 and D2 stand for the presence of DA receptors. Changes in parameters concern DA drive, expressed by $d_g$ in equation 1, and baselines for all units in the nuclei of STN and GP to simulate subthalamic DBS.
